# Improving Door to Groin Puncture Time for Mechanical Thrombectomy via Iterative Quality Protocol Interventions

**DOI:** 10.7759/cureus.2300

**Published:** 2018-03-10

**Authors:** Vincent J Cheung, Arvin R Wali, David R Santiago-Dieppa, Robert C Rennert, Michael G Brandel, Jeffrey A Steinberg, Brian R Hirshman, Kevin Porras, Peter Abraham, Julie Jurf, Emily Botts, Scott Olson, J. Scott Pannell, Alexander A Khalessi

**Affiliations:** 1 Department of Neurosurgery, University of California, San Diego

**Keywords:** door to groin puncture time, ischemic stroke, mechanical thrombectomy

## Abstract

Introduction: Delays in door to groin puncture time (DGPT) for patients with ischemic stroke caused by acute large vessel occlusions (LVO) are associated with worse clinical outcomes. We present the results of a quality improvement protocol for endovascular stroke treatment at the University of California, San Diego (UCSD) that aimed to minimize DGPT.

Materials and Methods: Our stroke team implemented a series of quality improvement measures to decrease DGPT, with a target of 90 minutes or less. Sixty-three patients treated at our center were retrospectively divided into three groups based on the date of their intervention as a proxy for the implementation of process improvement protocols: 23 patients treated from July to December 2015, 24 patients treated from January to July 2016, and 16 patients treated from July 2016 to December 2016. Multivariate log-linear and logistic regression analyses were used to assess the predictors of prolonged DGPT and compliance with target DGPT (<90 min), respectively.

Results: Date of intervention—a proxy for the implementation of process improvement protocols—was predictive of compliance with target DGPT. Patients treated from July 2016 to December 2016—after the full implementation of process improvements—were 3.2 times more likely to meet or exceed the target DGPT compared to patients treated from July 2015 to December 2015 (p=0.011). When adjusting for potential confounders in a multivariate analysis, patients in the final cohort were associated with shorter DGPT (Exp(B)=0.61, p=0.013) and remained significantly more likely to achieve the DGPT goal (OR=14.2, p=0.007).

Conclusion: An iterative quality improvement process can significantly improve DGPT. This analysis demonstrates the utility of a formal quality improvement system at an academic comprehensive stroke center.

## Introduction

Strokes are among the leading causes of global morbidity and mortality [1-2]. Large vessel strokes (or large vessel occlusions (LVO)) are especially dangerous and carry mortality or severe morbidity rates of up to 80% [3-4]. Mechanical thrombectomy (MT) for acute ischemic stroke due to LVO has demonstrated a dramatic clinical benefit in numerous randomized control trials [4-11] and is now the standard of care for these patients. The efficacy of MT for acute ischemic stroke is directly related to the timing of reperfusion, and delays in care can preclude intervention or result in worse clinical outcomes due to progressive, irreversible brain ischemia [7,12].

Current efforts to reduce the time from symptom onset to MT aim to optimize clinical outcomes in LVO, which mirror previous refinements in the interventional management of patients with acute myocardial infarction [13-14]. Sources of endovascular treatment delay for stroke can be broadly divided into 1) pre-hospital delays and 2) in-hospital delays [15-16]. Pre-hospital delays include failure to promptly identify symptoms of acute stroke, delays in transport due inadequate emergency medical services (EMS) infrastructure or weather, and limited regional availability of a stroke center with neurointerventional capabilities. As such, factors underlying pre-hospital delays are typically not within the direct control of a neurointerventional center and, instead, must be addressed at a regional level. Conversely, in-hospital delays include any portion of the neurointerventional activation process and, therefore, represent a significant opportunity to improve time to treatment. Prior studies in the management of ischemic stroke have reported institution-specific changes implemented to reduce in-hospital delays. Door-to-groin puncture time (DGPT) is the time required for a patient to reach the neuro-interventional radiology (IR) suite for mechanical thrombectomy [14,17-18]. In this study, we describe our experience with an 18-month quality improvement project targeted at reducing DGPT at an academic, comprehensive stroke center. 

## Materials and methods

In July 2015, we initiated a quality improvement (QI) project at the University of California San Diego (UCSD) Medical Center to reduce DGPT. Prior to the QI project, there was no formal workflow to activate the neurointerventional team for stroke treatment. We established a monthly stroke process improvement group that included representatives from endovascular neurosurgery, stroke neurology, stroke nursing, interventional radiology nursing, interventional radiology technologists, and neurocritical care. Cases from the preceding month were reviewed and points of delay were identified. Suggestions for improvement were developed into action items for implementation the next month. Additionally, key time points, such as time to reach the computed tomography (CT) scanner, time from CT scanner to the IR suite, door-to-tPA (tissue plasminogen activator), and door-to-groin puncture, were recorded. Interventions that failed to meet institutional time goals were automatically reviewed by the committee.

Cohort definitions and descriptions of outcomes

This retrospective study was approved by the institutional review board. Patients were included in this analysis if they underwent mechanical thrombectomy from July 2015 to December 2016. Patients under 18 years of age were excluded. In our analysis, the date of intervention was used as a proxy for the implementation of process improvement protocols to evaluate their impact on DGPT over time. Patients were divided into three groups based on the date of their intervention: Group 1 included 23 patients treated from July-December 2015, Group 2 included 24 patients treated from January-July 2016, and Group 3 included 16 patients treated from July 2016-December 2016. The successful execution of neurointerventional team activation was defined as the achievement of a target DGPT <90 minutes.

Statistical analysis

The statistical analysis was completed using R version 3.3.1 [19]. The achievement of the target DGPT (<90 minutes) was compared among the three different time groups. As the QI project was ongoing, Groups 1, 2, and 3 were deemed to have minimal, intermediate, and maximum implementation of process improvement measures, respectively. The univariate analysis was completed using Fisher’s exact test for assessing the achievement of the target DGPT (<90 min) and the Kruskal-Wallis test and the Wilcoxon rank-sum test for DGPT as a continuous variable. Multivariate log-linear regression was used to assess the predictors of prolonged DGPT. For this analysis, the dependent variable (DGPT) was log-transformed due to its nonparametric distribution. Multivariate logistic regression was used to assess compliance with target DGPT. In addition to the time period of intervention, covariates included mode of admission, admission shift (day vs. night), hospital location (location 1 vs. location 2), age, and sex. Statistical significance was defined as p≤0.05.

## Results

Patient characteristics

Sixty-three consecutive patients underwent MT for acute LVO from July 2015 to December 2016. The mean age was 69.1 years and 66.7% of the patients were male. The mode of arrival did not differ between groups (p>0.05). Overall, 38.1% of patients arrived via EMS; 28.6% were existing inpatients, 12.7% were admitted through the trauma system, 17.5% were transferred with an acute LVO diagnosed at an outside hospital, and 3.2% presented to the emergency department via private transport. Overall, 52.4% of patients presented during the hospital day shift (6:00 AM to 6:00 PM).

Quality improvement across time

**Figure 1:**
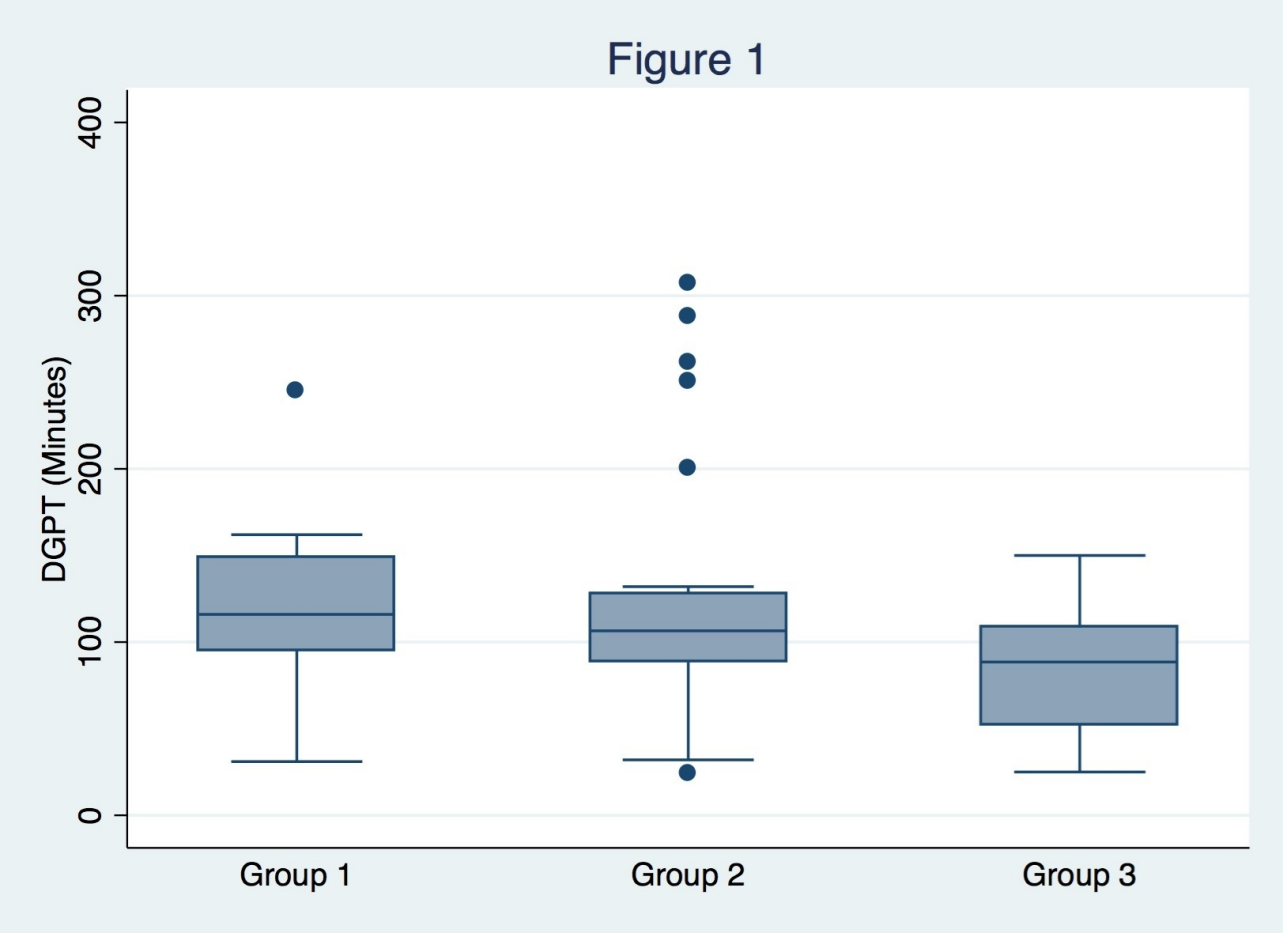
Change in door to groin puncture time in minutes across quality improvement measures Across the three cohorts, there was a decrease in the average DGPT that corresponded to the implementation of quality improvement measures. DGPT for Group 3 was significantly lower than DGPT for Group 1 (p=0.005).

**Figure 2:**
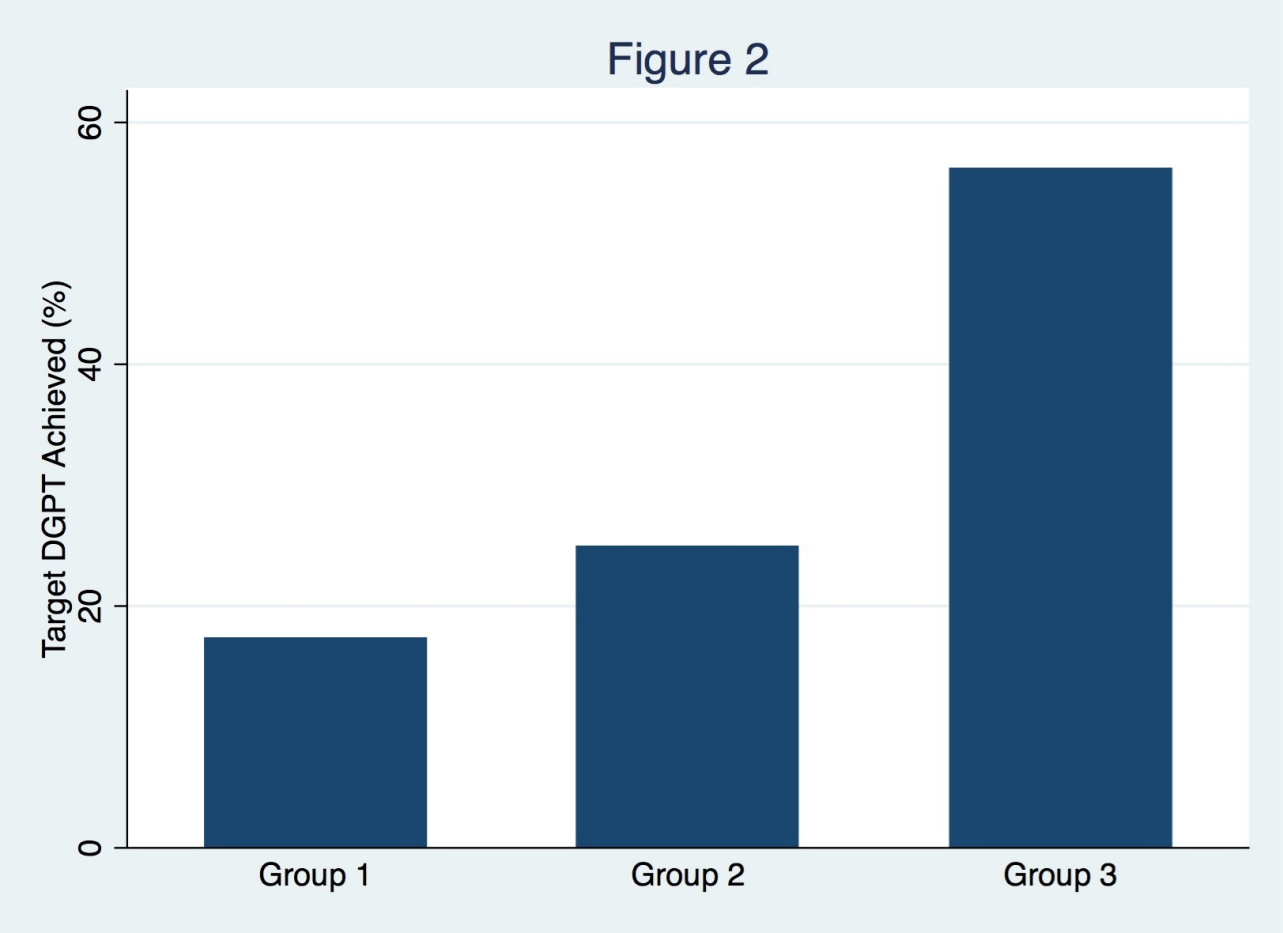
Change in the achievement of door to groin puncture time across intervention cohorts There was a significant increase in the proportion of patients achieving the DGPT goal of <90 minutes following the iterative implementation of quality improvement measures. Patients in Group 3 were 3.2 times more likely to achieve the target DGPT than subjects in Group 1 (p=0.012).

The implementation of QI measures was associated with an absolute reduction in DGPT. The median DGPT was 116 (interquartile range (IQR) 95-153) minutes in Group 1, 106.5 (IQR 88.5-128) minutes in Group 2, and 88.5 (IQR 52.5-108.5) minutes in Group 3 (p=0.023) (Figure 1). DGPT for Group 3 was lower than DGPT for Group 1 (p=0.005) but did not significantly differ between Groups 1 and 2 (p=0.282) or between Groups 2 and 3 (p=0.090). The achievement of target DGPT significantly differed between groups on univariate analysis (p=0.035) (Table 1). Four of 23 patients (17.4%) in Group 1, seven of 24 patients (29.2%) in Group 2, and nine of 16 patients (56.3%) in Group 3 achieved DGPT of less than 90 minutes (Figure 2). Subjects in Group 3 were 3.2 times more likely to achieve the target DGPT than subjects in Group 1 (p=0.012). When adjusting for potential confounders on multivariate log-linear regression, Group 3 remained associated with a significantly lower DGPT (Exp(B)=0.61, p=0.013).

**Table 1:** Baseline patient characteristics and results of the univariate analysis Abbreviations: *DGPT*, door to groin puncture time; *EMS*, Emergency Medical Services; *SD*, standard deviation; *IQR*, interquartile range

Table 1	Overall	Group			
		1	2	3	p
N	63	23	24	16	
Age, mean (SD)	69.13 (15.32)	63.65 (17.92)	73.25 (12.39)	70.81 (13.72)	0.086
Sex					0.069
Female	21 (33%)	9 (39%)	4 (17%)	8 (50%)	
Male	42 (67%)	14 (61%)	20 (83%)	8 (50%)	
Hospital Site					0.91
1	51 (81%)	18 (78%)	20 (83%)	13 (81%)	
2	12 (19%)	5 (22%)	4 (17%)	3 (19%)	
Mode of Arrival					0.90
EMS	24 (38%)	10 (43%)	7 (29%)	7 (44%)	
Inpatient	18 (29%)	7 (30%)	7 (29%)	4 (25%)	
Transfer	11 (17%)	3 (13%)	5 (21%)	3 (19%)	
Trauma	8 (13%)	3 (13%)	4 (17%)	1 (6%)	
Private Transport	2 (3%)	0 (0%)	1 (4%)	1 (6%)	
Arrival Time					0.39
Day	33 (52%)	14 (61%)	10 (42%)	9 (56%)	
Night	30 (48%)	9 (39%)	14 (58%)	7 (44%)	
DGPT, median (IQR)	107.00 (87.00, 134.00)	116.00 (95.00, 153.00)	106.50 (88.50, 128.00)	88.50 (52.50, 108.50)	0.022
Target DGPT					0.035
Not Achieved	43 (68%)	19 (83%)	17 (71%)	7 (44%)	
Achieved	20 (32%)	4 (17%)	7 (29%)	9 (56%)	

On multivariate logistic regression, Group 3 was significantly more likely to achieve the target DGPT compared to Group 1 (OR=14.2, p=0.007). The mode of arrival and the time of admission were also independently associated with the achievement of the DGPT goal. Patients arriving via transfer were associated with lower DGPT (Exp(B)=0.55, p=0.007) and were more likely to achieve target DGPT (OR=13.9, p=0.014) than patients arriving via other modes of transportation. Nighttime admissions were associated with higher DGPT (Exp(B)=1.41, p=0.023) and were less likely to achieve the target DGPT than daytime admissions (OR=0.19, p=0.034). The results of the multivariate analyses are shown in Table 2.

**Table 2:** Results of multivariate regression analyses Model 1, a multivariate log-linear regression analysis of DGPT in minutes. Model 2, a multivariate logistic regression analysis of achieving the target DGPT (<90 minutes). Abbreviations: *DGPT*, door to groin puncture time; *Exp(B)*, exponentiated regression coefficient; *OR*, odds ratio; *EMS*, Emergency Medical Services

Table 2	Model 1: DGPT		Model 2: Target DGPT	
	Exp(B)	p	OR	p
Group				
1 (Reference)	1	--	1	--
2	0.894	0.526	2.052	0.430
3	0.612	0.013	14.17	0.007
Age	1.002	0.675	0.969	0.232
Sex				
Female (Reference)	1	--	1	--
Male	0.767	0.121	4.114	0.141
Hospital Site				
1 (Reference)	1	--	1	--
2	0.953	0.816	1.099	0.931
Mode of Arrival				
EMS (Reference)	1	--	1	--
Inpatient	0.983	0.924	0.385	0.316
Transfer	0.548	0.007	13.90	0.014
Trauma	1.258	0.336	0.955	0.971
Private Transport	0.915	0.840	5.152	0.385
Arrival Time				
Day (Reference)	1	--	1	--
Night	1.408	0.023	0.187	0.034
Observations	63		63	

## Discussion

Current efforts to reduce DGPT mirror established refinements of myocardial infarction response systems following the introduction of emergent percutaneous coronary interventions [20-22]. Similar to the quality improvements made in the interventional cardiovascular field, our data demonstrate that iterative, team-based improvements to the process structures for stroke intervention can be used to improve quality-of care-metrics. In this project, all process changes were developed out of suggestions to address specific areas of inefficiency identified in real-life cases.

We observed that patients who presented during the day and as transfers had significantly faster pathways to the interventional suite. This likely stemmed from the immediate availability of the neurointerventionalist and interventional teams during the day, as well as the increased coordination time afforded by a patient being transferred with a known LVO. Many of the iterative improvements made in pathway processing attempted to recapitulate this ideal scenario. For instance, communication delays between the stroke neurology and neurointerventional surgery were identified as a frequent source of inefficiency. Though up-to-date call schedules were listed with the hospital operator, as a practical matter, the neurologists would typically consult the neurointerventional team by calling different surgeons until they found someone available. This contributed to suboptimal response times. The creation of a dedicated neurointerventional virtual pager provided a consistent "hotline" to the on-call neurointerventionalist. The development of a group alert system to activate the stroke-intervention team also helped streamline activation. Similarly, prior to our QI project, the on-call surgeon would page each member of the neurointervention team individually. This resulted in the surgeon communicating the same set of information multiple times. The creation of a "stroke intervention" paging group allowed simultaneous text communication to all members of the stroke intervention team and allowed all critical information to be relayed in a matter of seconds.

The electronic medical record also presented significant obstacles. Due to the coding of our institution's EMR, creating a record for a stroke case was initially extremely cumbersome. To help resolve this, we worked with our IT team and administration to remove technical roadblocks and, in case of transfer patients, prebooked the patient into our system while they were en route.

The consistent inclusion of an anesthesia team also markedly enhanced the workflow of our interventions. Initially, anesthesia was only requested on an ad hoc basis if the patient required intubation. The IR nurse and attending surgeon would be primarily responsible for conscious sedation anesthesia. However, in uncooperative or unstable patients, the simultaneous demands of airway management, medication administration, charting, and room circulation often overwhelmed the nurse, resulting in treatment delays. We found that delegating direct patient care to the anesthesia team streamlined this process.

Beyond the specific measures that were implemented, we found that the implementation of a formal QI process was empowering for all members of the multidisciplinary stroke team. Regardless of seniority, all suggestions were considered. This allowed our team to transform from a passive to an active quality improvement culture.

This study was performed retrospectively without historical case-matched controls. This was an intentional decision since prior to the QI project, our endovascular treatment strategies were not standardized among surgeons and many previous stroke treatments did not use latest-generation stent retriever devices. Since this study was confined to patients treated over an 18-month period, its power to elucidate absolute DGPT differences among subject groups was limited by sample size. Nonetheless, an absolute reduction in DGPT was observed with the progressive implementation of QI measures, and there was a significant increase in the proportion of patients meeting DGPT goals by the end of the QI project. These findings are similar to other published QI projects that demonstrated clearly defined tasks for members of the team and parallel workflows were critical to streamlining care in the interventional suite and thereby optimizing patient care [14,17].

## Conclusions

A formal quality improvement process is critical to optimizing response times for stroke intervention. While many challenges facing stroke centers may be institution-specific, general principles can be shared and adopted. Establishing parallel workflows and streamlining communication among teams is crucial to efficiency. Additionally, establishing set roles for each member of the team helps avoid redundancy and confusion. Quantifying outcome measures and analyzing performance to identify areas for optimization allows the rapid implementation and evaluation of process improvement measures. This quality improvement process can be easily translated to other comprehensive stroke centers with critical implications for the nationwide care of patients with large vessel strokes.
